# First molecular study in Lebanese patients with Cockayne syndrome and report of a novel mutation in *ERCC8* gene

**DOI:** 10.1186/s12881-018-0677-7

**Published:** 2018-09-10

**Authors:** Alain Chebly, Sandra Corbani, Joelle Abou Ghoch, Cybel Mehawej, André Megarbane, Eliane Chouery

**Affiliations:** 1 Medical Genetics Unit, Faculty of medicine, Saint Joseph University (USJ), Damascus street, B.P. 17-5208, Mar Mikhaël, Beirut, 1104 2020 Lebanon; 2 Institut Jérôme Lejeune, Paris, France

**Keywords:** Cockayne, CS, *ERCC8*, *ERCC6*, Sanger sequencing, Lebanon

## Abstract

**Background:**

Cockayne Syndrome (CS) is a rare autosomal recessive disorder characterized by neurological and sensorial impairment, dwarfism, microcephaly and photosensitivity. CS is caused by mutations in *ERCC6 (CSB)* or *ERCC8 (CSA)* genes.

**Methods:**

Three patients with CS were referred to the Medical Genetics Unit of Saint Joseph University. Sanger sequencing of both *ERCC8* and *ERCC6* genes was performed: *ERCC8* was tested in all patients while *ERCC6* in one of them.

**Results:**

Sequencing led to the identification of three homozygous mutations, two in *ERCC8* (p.Y322* and c.843 + 1G > C) and one in *ERCC6* (p.R670W). All mutations were previously reported as pathogenic except for the c.843 + 1G > C splice site mutation in *ERCC8* which is novel.

**Conclusions:**

Molecular diagnosis was established in all patients included in our study. A genotype-phenotype correlation is discussed and a link, between mutations and some specific religious communities in Lebanon, is suggested.

**Electronic supplementary material:**

The online version of this article (10.1186/s12881-018-0677-7) contains supplementary material, which is available to authorized users.

## Background

Cockayne syndrome (CS; MIM# 133540, 216400) is a rare autosomal recessive disorder belonging to the family of premature aging syndromes. It was first described by Edward Alfred Cockayne, a British physician, in 1936 in a paper entitled “Dwarfism with retinal atrophy and deafness”, followed ten years later by another paper reporting a follow up data on the same patients [1, 2]. The incidence of this syndrome is estimated to be 2.7 per million in Western Europe [3]. CS is a multisystem disorder characterized by growth failure, dwarfism, microcephaly, intellectual disability, senile face and photosensitivity [1, 4]. Other symptoms of the disease include retinopathy and hearing loss that are progressive during life and which severity correlates with the severity of the disease [4, 5]. Cataracts, for instance, found in almost 50% of CS patients, are associated with a poor prognosis when occurring at an early age [4, 6, 7]. CS is clinically divided into three subtypes: the classical form or CS type I, the severe form or CS type II and the mild form or CS type III [4, 8, 9]. The onset of symptoms in type I is in the early childhood, usually after one year of age. Type II, involving more severe symptoms, often exists at birth, while type III appears later in childhood [4, 10].

This rare disease is linked to mutations in one of two excision-repair cross-complementation genes *ERCC6* (*CSB)* and *ERCC8* (*CSA).* These genes encode proteins involved in the transcription-coupled sub-pathway of nucleotide excision repair (TC-NER) of UV-induced DNA damage [11, 12]. CS proteins are also implicated in the control of oxidative stress response and in the maintenance of mitochondrial function [13] . *ERCC6* (10q11.2) encodes CSB, a 1493 aa protein that belongs to the SNF2/SW12 ATPases family [14] while *ERCC8* (5q12.1) encodes CSA, a 396 aa protein, comprising WD (tryptophan-aspartic acid dipeptide) repeats. CSA interacts with CSB and p44, a subunit of the human RNA polymerase II transcription factor IIH [15, 16].

Among the reported CS cases, 62 to 68% had mutations in *ERCC6* gene [17, 18].

The diagnosis of CS in three Lebanese patients was confirmed, in 1999, by Jabre et al. using the recovery of RNA synthesis (RRS) assay after UVC irradiation in patients’ fibroblasts. Impaired RRS is typically found in CS cells. Molecular evaluation of these patients was not performed [19]. Here we report the first molecular study of CS in the Lebanese community.

## Methods

### Patients

Three patients with CS were referred to the Medical Genetics Unit of Saint Joseph University (USJ). Among them, two patients present a classical form and one a severe form. Approval to conduct this study was obtained from the Ethics Committee of Saint Joseph University (USJ), Beirut, Lebanon.

### Patient A

**Fig. 1:**
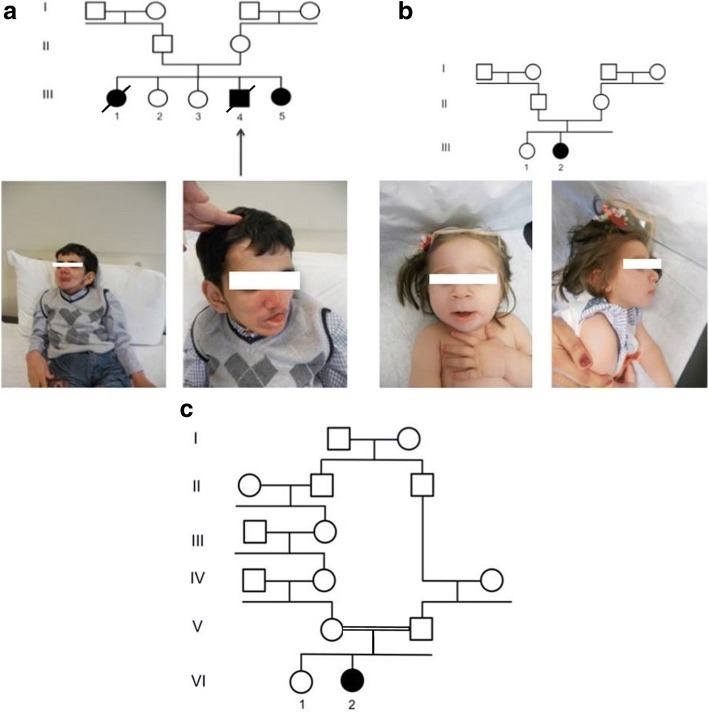
**a** Pedigree of family A and photos of patient A. **b** Pedigree of family B and photos of patient B. **c** Pedigree of family C

Patient A (Fig. 1a, patient III.4) is the fourth child born to non-consanguineous Christian (Greek Orthodox) parents, from the Akkar region in North Lebanon. Among the five children of this family, three were affected. Only patient III.4 presented to the clinical examination and was included in this study. He was born at term weighting 4 kg (90th Percentile). At 3 months of age, the parents noted a hypotonia. Patient A was first referred to us at the age of 16. His clinical examination showed hypotonia, a short stature (104 cm: < 3rd Percentile), a head circumference of 47 cm (< 3d Percentile) and a typical dysmorphic face associating microcephaly, enophtalmy, and an aquiline nose with a sensorial impairment including hearing loss. He had numerous dental caries and some skin lesions due to photosensitivity. Patient A deceased at 17 years of age.

### Patient B

Patient B (Fig. 1b, patient III.2) is the second child born to non-consanguineous Muslim parents, from Saida in South Lebanon. She was a term newborn weighting 3,1 kg (15th Percentile) with a height of 51 cm (50th Percentile) and a head circumference of 35,5 cm (75th Percentile). Patient B underwent two surgeries for bilateral congenital cataracts, one at the age of 2 months and the other at the age of 4 months. The parents noted the onset of cutaneous photosensitivity and skin lesions at an early age. Patient B presented to us at one year of age, with a height of 67 cm (< 3rd Percentile), a head circumference of 38,5 cm (<3rd Percentile) and a weight of 6.3 kg(< 3rd Percentile). Her clinical examination showed characteristic facial dysmorphisms including microcephaly, aquiline nose, low-set ears, deep-set eyes, dry skin and no teeth.

### Patient C

Patient C (Fig. 1c, patient VI.2) is the second child born to consanguineous Druze parents, from Kfarselwan in Mount Lebanon. She was delivered by cesarean section, weighting 2,9 kg (10th Percentile) with a height of 51 cm (50th Percentile) and a head circumference of 34 cm(35th Percentile). The onset of growth failure was first noted at the age of 3 months. Patient C was referred to us at the age of 2 years. Her clinical examination showed a remarkable growth failure in addition to some typical dysmorphic features including microcephaly, exophthalmia and an aquiline nose. At two and a half years old, patient B underwent a surgery for bilateral cataracts. At the age of 3 years, hearing loss started to manifest and led to deafness.

### Methods

#### DNA extraction and sanger sequencing

Peripheral blood samples were obtained from the three patients after obtaining written informed consent from the parents. Genomic DNA was isolated from white blood cells using standard salt-precipitation methods. Genomic sequence of *ERCC8* (NM_000082.3) and *ERCC6* (NM_000124.3) were obtained from UCSC Genomic Browser on Human (hg19).

PCR primers were designed using Primer3 software to amplify each of the 12 exons of *ERCC8* gene and the 21 exons of *ERCC6* gene as well as their flanking intronic sequences (Additional file 1: Table S1). PCR reactions were performed using Taq DNA polymerase (Invitrogen Life Technologies, Carlsbad, CA, USA). PCR fragments were run on 1% agarose gel. The fragments were purified using “SIGMA-ALDRICH™” GenElute PCR clean-up kit and then sequenced using Big Dye_ Terminator v1.1 Cycle sequencing kit (Applied Biosystems, Foster City, CA, USA). Sequence reaction was purified on Sephadex G50 (Amersham Pharmacia Biotech, Foster City, CA, USA) and loaded into an ABI 3500 Sequencer after the addition of Hidi formamide. Electropherograms were analyzed using Sequence Analysis Software version 5.2 (Applied Biosystems) and then aligned with the reference sequences using ChromasPro v1.7.6.1 (Technelysium, Queensland, Australia).

#### RNA extraction and cDNA sequencing

Peripheral blood samples were obtained from family C (patient C and parents) and RNA was isolated using Chomczynscki and Sacchi method with Trizol (Invitrogen Life Technologies, Carlsbad, CA, USA). Manual method with Phenol Chloroform was performed in order to obtain a better outcome. RT-PCR was used to obtain cDNA from RNA using SuperScript II reverse transcriptase (Invitrogen Life Technologies, Carlsbad, CA, USA) and then cDNA was treated like DNA.

In order to rule out contamination of RNA samples by genomic DNA, PCR of the housekeeping gene β-globin was performed using the following primers: Globin-F (5′-AAG TTG GTG GTG AGG CCC TG-3′) and Globin-R (5′-TTG CCA AAG TGA TGG GCC AG-3′). For each sample, two reactions were performed: the first allowing the amplification of the β-globin transcript (RT+) and the second allowing the exclusion of any contamination by genomic DNA (RT-) (Fig. 4c).

**Fig. 2:**
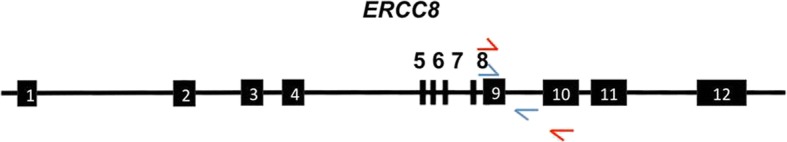
“Couple 1” (blue) and “couple 2” (red) of primers designed to amplify the exon9-intron 9 region of *ERCC8* gene

For *ERCC8* RT-PCR, two couples of primers were designed: Couple 1 with primer F in exon 9 and primer R in intron 9 to investigate if intron 9 is included in *ERCC8* mature transcript. Couple 2 with primer F in exon 9 and primer R in exon 10, which should amplify in a normal case. Both couples are shown in Fig. 2, couple 1 in blue and couple 2 in red.

## Results

The strategy adopted in this study consisted of initially sequencing exon 10 of the *ERCC8* gene based on the fact that the majority of mutations in CS patients from the Arab community are located in this exon [17]. If no mutations were detected, the rest of the *ERCC8* gene (11 exons) is tested. If *ERCC8* sequencing showed normal results, *ERCC6* is evaluated.

**Fig. 3:**
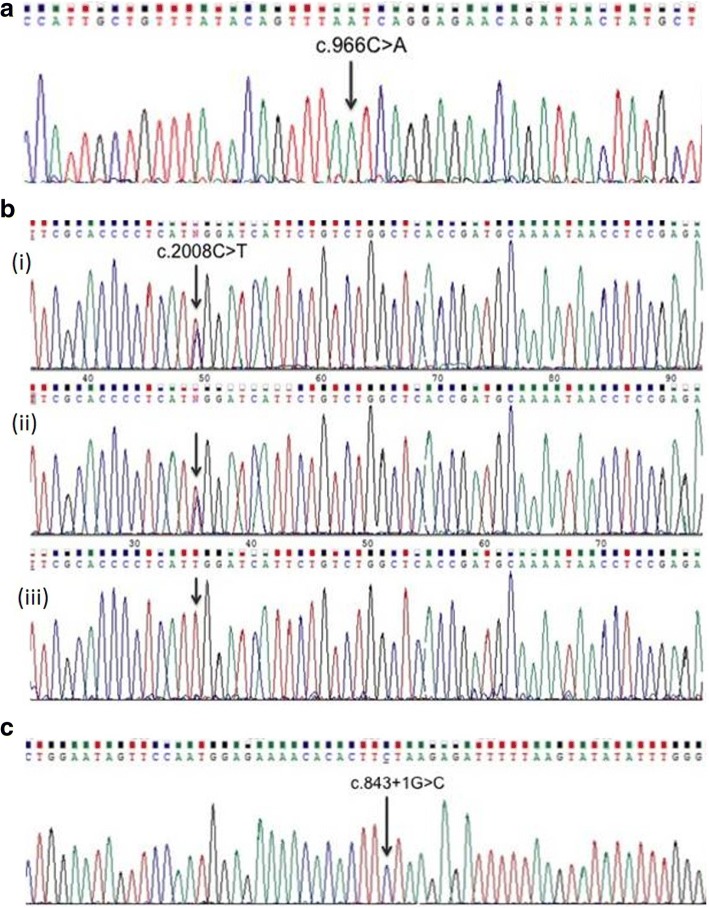
**a** Variation c.966C > A (p.Y322*) at homozygous state in the exon 10 of *ERCC8* gene in patient A. **b** Variation c.2008C > T (p.R670W) in *ERCC6* gene in family B: (i)Father, (ii)Mother and (iii)Patient B. **c** The novel variation c.843 + 1G > C in the junction exon9-intron9 of *ERCC8* gene in patient C

Sequencing led to the detection of a homozygous non-sense mutation in exon 10 of *ERCC8* (NM_000082.3:c.966C > A; p.Y322*) in patient A (Fig. 3a). Testing of *ERCC8* did not reveal any mutation in patient B, however, a homozygous mutation in exon 10 of the *ERCC6* gene (NM_000124.3:c.2008C > T; p.R670W) was detected in this patient (Fig. 3b). Parents of patient B are heterozygous for the same mutation (Fig. 3b). Genetic evaluation of patient C led to the identification of a novel homozygous variant in *ERCC8* (NM_000082.3:c.843 + 1G > C) altering the donor splice site of intron 9 of the gene (Fig. 3c).

**Fig. 4:**
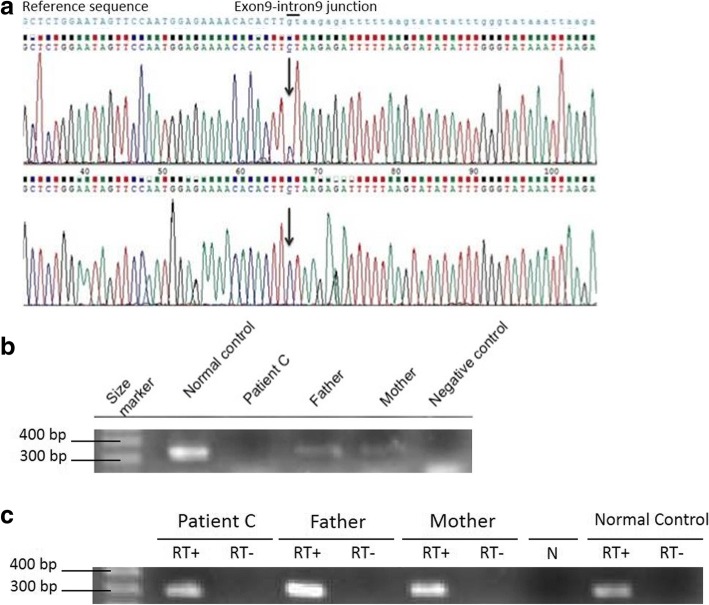
Patient C: **a** The result of sequencing after amplification of cDNA using “couple 1” of primers. **b** The results of amplification of cDNA (307 bp) using “couple 2” of primers. **c** β-globin PCR: two reactions were performed for each sample RT+ and RT-, and N is the negative control of the PCR

Interpretation of this novel variation using the prediction tool “Human Splicing Finder” revealed that its effect is most probably affecting the splicing. The effect of this splice site mutation was studied on RNA by PCR amplification using a couple of primers (couple 1) that includes a reverse primer specific to intron 9. Contamination with genomic DNA was ruled out using globin as a control (Fig. 4c). Amplification, using couple 1, was observed in patient C, thus suggesting that a part of intron 9 is included in *ERCC8*’s transcript. This result was confirmed by sequencing of the obtained amplicon (Fig. 4a).

The same PCR performed on the parents showed a weak amplification; hypothesizing that they carry one mutated copy of the *ERCC8* gene.

RNA Analysis using primers named “couple 2” showed no amplification for patient C and a normal amplification for a normal control. Absence of amplification is due to the large size of the amplicon including intron 9 in the mRNA (Fig. 4b). The same PCR performed for the parents showed poor amplification (307 bp) in comparison to the normal control (Fig. 4b) thus suggesting that the parents have one normal copy of *ERCC8* gene. Altogether, these data confirm the inclusion of at least a part of intron 9 in the mRNA of *ERCC8* (r.[843 + 1G > C; 843_844ins843 + 1_843 +?]), which is predicted to cause the insertion after the Leucine (281) of three amino acids (Arg, Asp, Phe) and a premature stop codon in CSB, thus mimicking the effect of the p.L281_V282insRDF* mutation.

In conclusion, three different mutations were identified in the Lebanese CS patients included in this study: one in *ERCC6* and two in *ERCC8*.

## Discussion

Three Lebanese patients originating from different geographic regions and belonging to different religious communities were included in this study. Of these patients, only one (patient C) is born to consanguineous parents.

All CS patients, including the three patients herein reported, present microcephaly, growth failure, typical face, intellectual disability and photosensitivity. In addition to these symptoms, Patient B has bilateral congenital cataracts and is affected by the severe form of the disease CSII, based on Lowry’s classification [8]. Patients A and C, who showed a normal intra uterine growth and symptoms that appeared around the age of 3 months, are affected with the classical form of the disease: CSI.

In order to identify the molecular basis of CS in these patients, *ERCC8* and *ERCC6* genes were analyzed: two different mutations in *ERCC8* (p.Y322* and c.843 + 1G > C) were detected in two patients and one in *ERCC6* (p.R670W) in the third.

The nonsense mutation p.Y322* (c.966C > A) in the *ERCC8* gene was found at a homozygous state in patient A who presents with CS type I, which correlates with a published genotype-phenotype study showing that the majority of patients with *ERCC8* mutations (75% of CSA patients) are classified as CSI [10]. Since consanguinity is not reported in family A and parents of patient A were not analyzed, the occurrence of a large genomic deletion of one *ERCC8* allele cannot be completely ruled out. The identified mutation was previously linked to CS in Christian Arabs communities and in patients from a Lebanese origin [17]. The detection of the same mutation in patient A who is born to a Christian Lebanese family is in support of the published data. Altogether, these findings might guide the diagnosis strategy for CS patients belonging to the Christian Arabs communities towards the prioritization of the sequencing of exon 10 in *ERCC8*.

The missense mutation p.R670W (c.2008C > T) in *ERCC6* was found at a homozygous state in patient B who is clinically classified as CSII. Patient B thus belongs to the 56% of patients with *ERCC6* mutations (CSB patients) who were shown to present with CSII [10]. The same mutation was found in a CS patient with Caucasian origins who presents with CSI, with an age of onset of a year and a half and without cataracts [17]. The absence of genotype-phenotype correlation, in this case, might be due to the fact that the Caucasian patient is compound heterozygous for this mutation.

The novel mutation c.843 + 1G > C in *ERCC8* was found at a homozygous state in patient C who presents with CSI as the majority of CSA patients [10]. The identified mutation affects the splicing donor site of intron 9 of *ERCC8*. RNA studies and analysis confirmed the inclusion of intron 9 in the mRNA of the patient, which alters the protein sequence. The c.843 + 1G > C variation is predicted to lead to a premature stop codon, thus mimicking a p.V282Lfs*5 mutation. Two different mutations affecting the splicing of exon 9 were already described: the first affecting the donor splice site (c.843 + 2 T > C) and the second (c.843 + 5G > C) predicted to lead to a premature stop codon mimicking a p.A240Gfs*8 mutation [17, 20]. However, in both studies RNA analysis were not performed.

All identified mutations are homozygous, which raises the possibility of the presence of a common ancestor per family, especially that couples of the non-consanguineous families A and B are from the same village and of the same religion. In fact, consanguineous marriages and same-religion marriages are widely practiced in the Middle East region and particularly in Lebanon where the population is divided into different religious groups. Jalkh et al.*,* showed a high prevalence of rare recessive diseases in offspring of related and unrelated couples in Lebanon, which is concordant with the data presented in our study [21].

## Conclusions

In conclusion, this is the first molecular study of CS in Lebanon. Here we report mutations in *ERCC8* and *ERCC6*, of which one is a novel splice site variation altering the splicing of *ERCC8*.

The finding of specific mutations in some religious communities suggests the possibility of the existence of a link between the clustering of mutations and the community to which belong a CS patient. Further molecular studies are needed to confirm this hypothesis.

Our study showed a concordance with the trend linking between the severity of CS and the molecular basis of the disease. However, a clear genotype – phenotype correlation has yet to be established in order to pave the way for a rapid molecular diagnosis.

## Additional file

Additional file 1: **Table S1.** List of primers used to amplify the exons of *ERCC8* and *ERCC6* genes. (DOCX 19 kb)

## Abbreviations

CS: Cockayne Syndrome
CSA: Cockayne Syndrome A
CSB: Cockayne Syndrome B
*ERCC6*: Excision Repair Cross-Complementation group 6
*ERCC8*: Excision Repair Cross-Complementation group 8
UVC: Ultra Violet C

## References

[1] Cockayne EA (1936). Dwarfism with retinal atrophy and deafness. Arch Dis Child.

[2] Cockayne EA (1946). Dwarfism with retinal atrophy and deafness. Arch Dis Child.

[3] Kleijer WJ, Laugel V, Berneburg M, Nardo T, Fawcett H, Gratchev A (2008). Incidence of DNA repair deficiency disorders in western Europe: Xeroderma pigmentosum. Cockayne syndrome and trichothiodystrophy DNA Repair.

[4] Nance MA, Berry SA (1992). Cockayne syndrome: review of 140 cases. Am J Med Genet.

[5] Gandolfi A, Horoupian D, Rapin I, DeTeresa R, Hyams V (1984). Deafness in Cockayne’s syndrome: morphological, morphometric, and quantitative study of the auditory pathway. Ann Neurol.

[6] Natale V (2011). A comprehensive description of the severity groups in Cockayne syndrome. Am J Med Genet A.

[7] Wilson BT, Stark Z, Sutton RE, Danda S, Ekbote AV, Elsayed SM (2016). The Cockayne syndrome natural history (CoSyNH) study: clinical findings in 102 individuals and recommendations for care. Genet med off J am Coll. Med Genet.

[8] Lowry RB (1982). Early onset of Cockayne syndrome. Am J Med Genet.

[9] Sugita K, Takanashi J, Ishii M, Niimi H (1992). Comparison of MRI white matter changes with neuropsychologic impairment in Cockayne syndrome. Pediatr Neurol.

[10] Laugel V (2013). Cockayne syndrome: the expanding clinical and mutational spectrum. Mech Ageing Dev.

[11] de Boer J, Hoeijmakers JH (2000). Nucleotide excision repair and human syndromes. Carcinogenesis.

[12] van Hoffen A, Balajee AS, van Zeeland AA, Mullenders LHF (2003). Nucleotide excision repair and its interplay with transcription. Toxicology.

[13] D’Errico M, Pascucci B, Iorio E, Van Houten B, Dogliotti E (2013). The role of CSA and CSB protein in the oxidative stress response. Mech Ageing Dev.

[14] Lake RJ, Fan H-YSTRUCTURE (2013). Function and regulation of CSB: a multi-talented gymnast. Mech Ageing Dev.

[15] Henning KA, Li L, Iyer N, McDaniel LD, Reagan MS, Legerski R (1995). The Cockayne syndrome group a gene encodes a WD repeat protein that interacts with CSB protein and a subunit of RNA polymerase II TFIIH. Cell.

[16] Saijo M (2013). The role of Cockayne syndrome group a (CSA) protein in transcription-coupled nucleotide excision repair. Mech Ageing Dev.

[17] Laugel V, Dalloz C, Durand M, Sauvanaud F, Kristensen U, Vincent MC (2010). Mutation update for the CSB/ERCC6 and CSA/ERCC8 genes involved in Cockayne syndrome. Hum Mutat.

[18] Calmels N, Botta E, Jia N, Fawcett H, Nardo T, Nakazawa Y (2018). Functional and clinical relevance of novel mutations in a large cohort of patients with Cockayne syndrome. J Med Genet.

[19] Jabre P, Mezzina M, Megarbane A (1999). Cockayne syndrome in Lebanon. Description of 3 cases and review of the literature. J Med Liban.

[20] Wang X, Huang Y, Yan M, Li J, Ding C, Jin H, et al. Molecular spectrum of excision repair cross-complementation group 8 gene defects in Chinese patients with Cockayne syndrome type A. Sci Rep. 2017;7(1). 10.1038/s41598-017-14034-3 PMC5651726 29057985

[21] Jalkh N, Sahbatou M, Chouery E, Megarbane A, Leutenegger A-L, Serre J-L (2015). Genome-wide inbreeding estimation within Lebanese communities using SNP arrays. Eur J Hum Genet.

